# Age-specific prevalence of serrated lesions and their subtypes by screening colonoscopy: a retrospective study

**DOI:** 10.1186/1471-230X-14-82

**Published:** 2014-04-28

**Authors:** Hyun Young Kim, Seon Mie Kim, Ji-Hyun Seo, Eun-Ha Park, Nayoung Kim, Dong Ho Lee

**Affiliations:** 1 Department of Internal Medicine, Seoul National University Bundang Hospital, Seongnam-si, Gyeonggi-do, Korea; 2 Department of Internal Medicine, Seoul National University College of Medicine, Seoul, Korea

**Keywords:** Colonic polyp, Serrated lesions, Prevalence, Screening colonoscopy

## Abstract

**Background:**

Serrated lesions of the colorectum as categorized by pathology include hyperplastic polyps, sessile serrated adenomas without dysplasia, and traditional serrated adenomas with dysplasia. The aim of this study was to investigate the prevalence of various subtypes of serrated lesions by age.

**Methods:**

In this study, 28,544 consecutive asymptomatic patients (aged 22–88 years) were evaluated during health check-ups involving colonoscopies performed by gastroenterologists at a single institution from 2005 to 2012.

**Results:**

The adenoma detection rate during colonoscopies for patients aged ≥50 years was 31.8% (25.0–35.8%). The serrated lesion detection rate for patients aged ≥50 years was 15.3% (10.5–19.6%). Serrated lesions were detected in 15.1% of all patients with subtype prevalences of 14.7% for hyperplastic polyps, 0.5% for sessile serrated adenomas, and 0.1% for traditional serrated adenomas. The prevalence of conventional adenomas increased sharply with age (5.0% in patients aged 20–29 years, 10.9% in those aged 30–39 years, 21.8% in those aged 40–49 years, 29.5% in those aged 50–59 years, 36.9% in those aged 60–69 years, and 40.7% in those aged ≥70 years) (trend *P* = 0.027). In contrast, the prevalence of serrated lesions increased only slightly with age (10.0% in patients aged 20–29 years, 11.8% in those aged 30–39 years, 14.8% in those aged 40–49 years, 15.3% in those aged 50–59 years, 16.8% in those aged 60–69 years, and 16.4% in those aged ≥70 years) (trend *P* = 0.036).

**Conclusions:**

The screening colonoscopy detection rate of serrated lesions, including sessile serrated adenomas and traditional serrated adenomas, appears to be relatively high among young patients aged <50 years.

## Background

The incidence and mortality rates associated with colorectal cancer are rapidly rising in Korea [1]. The current Korean guidelines recommend the performance of screening colonoscopy beginning at age 50 years in the average-risk population [2]. Screening colonoscopy reportedly facilitates early detection and prevention of colorectal cancer that develops from the adenoma–carcinoma pathway [3,4]. However, right-sided colorectal cancer that develops from the serrated pathway may be less effectively detected by screening colonoscopy [5].

Serrated lesions of the colorectum are classified into three heterogeneous categories according to the World Health Organization: conventional hyperplastic polyps, sessile serrated adenomas, and traditional serrated adenomas [6]. Serrated lesions have a distinct endoscopic appearance. Sessile serrated adenomas are flat or sessile, poorly demarcated, and waxy or pale, and they may be covered with a mucus cap. Traditional serrated adenomas are occasionally peduculated. The detection rate of serrated lesions is closely dependent on the endoscopist.

An effective colonoscopy is necessary for early diagnosis of colorectal cancer precursor lesions, adenomas, or serrated lesions. The adenoma detection rate has been validated by endoscopists as a predictor of interval cancer and a surrogate indicator of the quality of screening colonoscopy [7,8]. Wide variability in the adenoma detection rate exists among endoscopists in previous studies [9,10], and the adenoma detection rate has also shown a strong correlation with serrated lesion detection rate. Moreover, a recent study showed that the serrated lesion detection rate is an important indicator of the quality of colonoscopy [11].

The aim of this study was to investigate the prevalence of various serrated lesion subtypes according to age and assess the variability in the serrated lesion detection rate among expert endoscopists.

## Methods

### Study population

The study population comprised all patients who underwent colonoscopies in a single tertiary hospital (Seoul National University Bundang Hospital) from January 2005 to August 2012. In total, 28,544 asymptomatic patients aged 22 to 88 years at average risk for colorectal cancer underwent a complete screening colonoscopy. All patients filled out a questionnaire regarding their family history of colorectal cancer, physical activity, alcohol drinking habits, smoking habits, and hormone use. With respect to smoking, each patient was categorized as a never-smoker, former smoker, or current smoker. Seoul National University Bundang Hospital Health Promotion Center provided the various examination packages that were required, including that for colonoscopy. All screened patients underwent colonoscopy on a volunteer or employer-sponsored basis regardless of age; the most important issue among the study population was the cost of colonoscopy ($US 60 in Korea). This study was approved by the Institutional Review Board of Seoul National University Bundang Hospital.

### Colonoscopy

Four expert gastroenterologists (>1,000 colonoscopies) who were certified by the Korean Society of Gastrointestinal Endoscopy and one nonexpert endoscopist (<300 colonoscopies) performed all endoscopies (CF-Q260AI/AL; Olympus, Tokyo, Japan) with images displayed on standard-definition video monitors. Patients were excluded from the study if they had hereditary polyposis syndrome, inflammatory bowel disease, or an incomplete study. Bowel preparations comprised 4 L of polyethylene glycol-based or sodium phosphate-based solution. The proximal colon was defined as that portion proximal to the splenic flexure (transverse colon, ascending colon, cecum, and ileocecal valve). Polyps were pathologically defined as adenomas, serrated lesions, or carcinoid tumors. Adenomas were classified as tubular, tubulovillous, or villous adenomas with low- to high-grade dysplasia or as adenocarcinomas. Serrated lesions were classified as hyperplastic polyps, sessile serrated adenomas, or traditional serrated adenomas. Advanced adenomas were defined as large adenomas (≥10 mm in size); adenomas with histopathological findings of tubulovillous, villous, or high-grade dysplasia; or adenocarcinoma. Endoscopists used the open-biopsy forceps method to estimate the size of the polyp or measure the actual size of the polyp after removal during colonoscopic polypectomy.

### Statistical analyses

Data analyses were performed using SPSS software (version 18.0; SPSS Inc., Chicago, IL, USA). Continuous variables are expressed as mean ± standard deviation, while categorical variables are expressed as absolute values and percentages. Continuous variables are presented as medians and ranges, and categorical variables are presented as percentages. Differences between variables were assessed by the χ^2^ test. All *P* values were two-sided, and a *P* value of <0.05 was considered statistically significant.

## Results

This study included 28,544 patients (17,357 [60.8%] men; mean age, 52 ± 10 years) who underwent health check-ups in a single endoscopy unit for screening colonoscopy. In total, 18,689 polyps were found in 10,358 of the 28,544 patients (36.3% of patients). Among these polyps, 71.4% were proximal polyps and 5.0% were large polyps (≥10 mm). Serrated lesions accounted for 42% of all colonic polyps. Table 1 shows the differences in demographics and clinical characteristics between the patients with adenomatous polyps and those with serrated lesions. The patients with serrated lesions were more likely to be young, smokers (current or former), and alcohol drinkers (*P* < 0.001).

**Table 1:** Demographics and baseline characteristics of patients

	**Any adenoma**	**Any serrated lesions**	**Adenoma & SL**	***P *value**
Subjects, n	7775 (27.2%)	4312 (15.1%)	1750 (6.1%)	
Age, mean, range, years	55.2 (25–88)	51.4 (22–83)	55.5 (27–82)	<0.001
Male,%	67.7	67.9	81.8	<0.001
Current or ex-smokers,%	59.7	65.1	76.5	<0.001
Alcohol use,%	71.8	75.5	21.3	<0.001
Hormone use,%	2.1	2.0	1.0	0.011
Family history of CRC,%	42.8	41.0	43.1	0.256

*CRC* colorectal cancer, *SL* serrated lesions.

### Adenoma and serrated lesion detection rates by endoscopists

Table 2 depicts the prevalence of adenomas and serrated lesions according to pathology, location, and size by colonoscopy. In total, 7,830 adenomas were detected in 28,544 patients (27.4%), and 51 colorectal cancers were detected (0.2%). The adenoma detection rate among patients of all ages was 27.4%, whereas that in patients aged ≥50 years was 31.8%.

**Table 2:** Polyp prevalence according to location and size by colonoscopy

**Polyp histology**	**Subjects**	**Location (%)**		**Size**		
	**10358/28544**	**Proximal (58.2%)**	**Distal (41.8%)**	**≤9 mm**	**10-20 mm**	**>20 mm**
Adenoma	7830 (27.4%)	5150 (65.8%)	4904 (62.6%)	7745 (98.9%)	311 (4.0%)	29 (0.4%)
Tubular, low grade	7749 (27.1%)	5139 (66.3%)	4888 (63.1%)	7736 (99.8%)	302 (3.9%)	20 (0.3%)
Tubular, high grade	19 (0.1%)	16 (84.2%)	14 (73.7%)	19 (100%)	1 (5.3%)	1 (5.3%)
Villous/tubulovillous	11 (0.03%)	8 (72.7%)	9 (81.8%)	10 (90.9%)	5 (45.5%)	0
Adenocarcinoma	51 (0.2%)	31 (60.8%)	42 (82.4%)	35 (68.6%)	21 (41.2%)	19 (37.3%)
Serrated lesions	4312 (15.1%)	2241 (52.0%)	3444 (79.9%)	4309 (99.9%)	166 (3.8%)	9 (0.2%)
Hyperplastic	4187 (14.7%)	2141 (51.1%)	3395 (81.1%)	4185 (99.9%)	162 (3.9%)	9 (0.2%)
Sessile serrated adenoma	143 (0.5%)	123 (86.0%)	66 (46.2%)	142 (99.3%)	9 (6.3%)	0
Traditional serrated adenoma	17 (0.1%)	10 (58.8%)	14 (82.4%)	17(100%)	2 (11.8%)	0
Carcinoid	26 (0.1%)	4 (15.4%)	25 (96.2%)	24 (92.3%)	1 (3.8%)	1 (3.8%)

Table 3 depicts the adenoma and serrated lesion detection rates by endoscopists. The adenoma detection rate among patients of all ages was significantly correlated with the serrated lesion detection rate (*R* = 0.94, *P* = 0.020). There was a significant correlation between the adenoma and serrated lesion detection rates for patients aged >50 years (*R* = 0.93, *P* = 0.022). The highest adenoma detection rate among all endoscopists was 35.8% in patients aged ≥50 years. The adenoma detection rate differed between the four expert endoscopists (31.4%–35.8%) (endoscopists A, B, C, and D) and the nonexpert endoscopist (25.0%) (endoscopist E). Adenomas were proximal in 5,150 (65.8%) patients and distal in 4,904 (62.6%) patients. The highest serrated lesion detection rate was 19.6% in patients aged ≥50 years. The overall serrated lesion detection rate was 15.3%. In patients aged ≥50 years, the serrated lesion detection rate ranged from 14.5% to 19.6% among the four expert endoscopists and the nonexpert endoscopist (10.5%).

**Table 3:** Adenoma and serrated lesion detection rates by endoscopist

**Endoscopist**	**n**	**ADR for all subjects (%)**	**SDR for subjects (%)**	**ADR for all subjects (≥50 years) (%)**	**SDR for subjects (≥50 years) (%)**
A	6805	30.0	16.6	35.8	17.1
B	6461	26.9	13.5	31.4	14.6
C	5965	29.5	18.8	34.9	19.6
D	6757	26.2	14.6	31.8	14.5
E	2556	20.4	9.7	25.0	10.5
mean	28544	27.4 (20.4-30.0)	15.1 (9.7-18.8)	31.8 (25.0-35.8)	15.3 (10.5-19.6)
*R*, *P* value		*R* = 0.94, *P* = 0.020		*R* = 0.93, *P* = 0.022	

*ADR* adenoma detection rate, *SDR* serrated lesion detection rate, *R* Pearson’s correlation coefficient.

### Serrated lesion subtype detection rate and prevalence by age decade

In total, 4,312 serrated lesions were detected in 28,544 patients (15.1%). The hyperplastic polyp prevalence rate was 14.7% (n = 4,187), the sessile serrated adenoma prevalence rate was 0.5% (n = 143), and the traditional serrated adenoma prevalence rate was 0.1% (n = 17). Serrated lesions were proximal in 2,241 (52.0%) and distal in 3,444 (79.9%) patients. Subtype analysis revealed that more hyperplastic polyps (81.1%) and traditional serrated adenomas (82.4%) were located in the distal colon, while more sessile serrated adenomas (86.0%) were located in the proximal colon. Table 4 shows the serrated lesion prevalence by age category and sex. There was a steady trend in the prevalence of serrated lesions with increasing age (trend *P* = 0.036) and an increasing trend in the prevalence of adenomas with age (trend *P* = 0.027). The serrated lesion prevalence in patients aged 20 to 29 years was 10.0% (22/219), in those aged 30 to 39 years was 11.8% (300/2548), in those aged 40 to 49 years was 14.8% (1323/8960), in those aged 50 to 59 years was 15.3% (1529/9994), in those aged 60 to 69 years was 16.8% (888/5296), and in those aged >70 years was 16.4% (250/1527).

**Table 4:** Polyp prevalence by age and sex

**Polyp histology**	**Subjects**	**Male (%)**	**Age**						**Trend *P***
	**10358/28544**		**20-29 (n = 219)**	**30-39 (n = 2548)**	**40-49 (n = 8960)**	**50-59 (n = 9994)**	**60-69 (n = 5296)**	**≥70 (n = 1527)**	
Adenoma	7775 (27.2%)	5511 (70.9)	11 (5.0%)	279 (10.9%)	1954 (21.8%)	2958 (29.5%)	1952 (36.9%)	621 (40.7%)	0.027
Tubular, low grade	7749 (27.1%)	5498 (71.0)	11	279	1951	2948	1940	620	
Tubular, high grade	19 (0.1%)	10 (50)	0	0	1	8	7	3	
Villous/tubulovillous	11 (0.03%)	11 (100)	0	1	2	2	3	3	
Adenocarcinoma	51 (0.2%)	36 (70.6)	0	0	5	14	26	6	
Serrated lesions	4312 (15.1%)	3170 (73.5)	22 (10.0%)	300 (11.8%)	1323 (14.8%)	1529 (15.3%)	888 (16.8%)	250 (16.4%)	0.036
Hyperplastic	4187 (14.7%)	3097 (74.0)	21	287	1277	1494	866	242	
Sessile serrated	143 (0.5%)	86 (60.1)	1	18	50	45	29	5	
Traditional serrated	17 (0.1%)	13 (76.5)	0	2	6	2	4	3	
Carcinoid	26 (0.1%)	17 (65.4)	0	6	11	7	2	0	

## Discussion

The present cross-sectional analysis of serrated lesions of the colorectum at a single institution revealed the prevalence of various subtypes of serrated lesions by patient age and elucidated the detection rates among endoscopists during screening colonoscopies. The serrated lesion subtype prevalence in average-risk patients undergoing screening colonoscopy in the present study was similar to that previously reported. Serrated lesions were detected in 15.1% of patients, including 14.7% hyperplastic polyps, 0.5% sessile serrated adenomas, and 0.1% traditional serrated adenomas. Compared with a recently published study [9] with a detection rate of 11.7% for hyperplastic polyps, 0.6% for sessile serrated adenomas, and 0.2% for traditional serrated adenomas, our results emphasize the effect of screening colonoscopy on the detection rate of serrated lesion subtypes among gastroenterologists in a general population-based setting of young to old patients (range, 22–88 years of age). The age-specific prevalence of serrated lesions steadily increased with age, while that of conventional adenomas sharply increased with age.

Colonoscopy with polypectomy significantly reduces the risk of death from colorectal cancer compared with the general population [6,12]. Colonoscopy does not reduce the incidence of death caused by right-sided colorectal cancer [13]. However, recent studies showed that a long-term effect of colonoscopy and a modest risk reduction for proximal colon cancer was achieved by colonoscopy in a United States cohort [5,14] and German cohort [15]. Colonoscopy performed by a gastroenterologist was more protective against colorectal cancer mortality than was colonoscopy performed by other providers.

The adenoma detection rate of ≥20% (≥25% in men ≥50 years of age and ≥15% in women ≥50 years of age) during screening colonoscopy, which was developed as a quality indicator in 2002 [7], has now been validated as a powerful predictor of the colorectal cancer risk after screening colonoscopy. In a Polish study [8], the adenoma detection rate was associated with the risk of interval cancer during screening colonoscopy. However, 37.5% of those with adenoma detection rates of <11% had colonoscopic experience of >10 years and 43% of those with adenoma detection rates <11% for all endoscopists.

One editorial offered several potential explanations and possible solutions for the relatively poor protection offered by colonoscopy against right-sided colon cancer [16]. Some of these explanations included poor proximal colon protection in the form of poor bowel preparation, incomplete cecal intubation, failed detection of flat or depressed lesions, and failed detection of serrated lesions. These could be addressed by split dose preparation, documentation by landmarks, measurement of adenoma detection rates, measurement of serrated lesion detection rates, and education on detection of proximal colon serrated lesions. An ASGE/ACG Taskforce on Quality in Endoscopy proposed that effective endoscopists should achieve a cecal intubation rate of ≥90% of all cases and ≥95% of screening colonoscopies [17].

Another explanation for the relatively poor protection offered by colonoscopy against right-sided colon cancer is the continuum of molecular changes (*CIMP*, *MSI*, and *BRAF* mutations) from the rectum to the ascending colon. This study has a substantial impact on the field of gastroenterology because of the prevalent dogma of proximal versus distal dichotomy, which is clearly an oversimplification [18,19].

The serrated lesion detection rate has a wide range that is dependent on the endoscopist’s experience and method. Two recent retrospective studies have evaluated the serrated lesion detection rate in average-risk patients aged ≥50 years during screening colonoscopy [9,11]. In a 2010 study [9], 7,192 colonoscopies at a single center were stratified by 13 endoscopists. The hyperplastic polyp detection rate ranged from 7.7% to 31.0%, the sessile serrated adenoma detection rate ranged from 0.0% to 2.2%, and the traditional serrated adenoma detection rate ranged from 0.0% to 0.5%. Additionally, a 2012 study [11] described 6,681 colonoscopies performed by 15 endoscopists. The proximal serrated lesion detection rate ranged from 1.0% to 18%. Our study showed a mean proximal serrated lesion detection rate of 7.9% with a narrowly ranged serrated lesion detection rate of 14.5% to 19.6% among the four expert gastroenterologists. However, the serrated lesion detection rate was wide when the nonexpert gastroenterologist was included (10.5%–19.6%). This suggests the effect of the endoscopist’s experience. The four expert endoscopists’ numbers of years of experience were very similar since gastroenterology fellowship graduation (range, 9–10 years).

Our data demonstrate that serrated lesions tend to develop more frequently than conventional adenomas in younger patients (aged 20–39 years). The prevalence of conventional adenomas increased sharply with age (5.0% in patients aged 20–29 years, 10.9% in those aged 30–39 years, 21.8% in those aged 40–49 years, 29.5% in those aged 50–59 years, 36.9% in those aged 60–69 years, and 40.7% in those aged ≥70 years). In contrast, the prevalence of serrated lesions increased only slightly with age (10.0% in patients aged 20–29 years, 11.8% in those aged 30–39 years, 14.8% in those aged 40–49 years, 15.3% in those aged 50–59 years, 16.8% in those aged 60–69 years, and 16.4% in those aged ≥70 years). This difference in the age-specific prevalence between serrated lesions and conventional adenomas may be due to several factors. For example, as many as 15% of colorectal cancers occurred in patients <50 years of age, which is the age at which we routinely start performing screening colonoscopy for colon cancers. Additionally, serrated lesions may be contributors to 15% to 35% of cases of colorectal cancer development through the serrated polyp–carcinoma pathway [3,4] and to the majority of cases of interval cancer development [20].

A strength of the current study is that it is the first to include patients in a young age category (<50 years of age) for evaluation of the serrated lesion prevalence using colonoscopy and precise categorization of the subtypes of serrated lesions. Importantly, it is the largest such study to date, comprising 28,544 patients who underwent screening colonoscopy to determine the age-specific prevalence of serrated lesions. An additional strength is that the majority of colonoscopies were performed by highly experienced endoscopists, producing high-quality data. All screened patients answered a questionnaire prior to colonoscopy, suggesting minimum recall bias. However, this study also has some limitations. Its main limitation is its cross-sectional, retrospective design, which introduces bias and leads to underestimation of the prevalence of small, left-sided hyperplastic polyps. A second limitation is the use of standard-definition white-light colonoscopes. Whether there is a significant difference between the detection rates of high- and standard-definition white-light colonoscopy remains unclear. A third limitation is that observer variation among pathologists in the diagnosis of serrated lesions could lead to underestimation of the true prevalence of sessile serrated adenomas and traditional serrated adenomas and thus to overestimation of the true prevalence of hyperplastic polyps.

## Conclusions

The prevalence of serrated lesions, including sessile serrated adenomas and traditional serrated adenomas, appears to be relatively high among young patients aged <50 years by routine screening colonoscopy.

## Competing interests

The authors declare that they have no competing interests.

## Authors’ contributions

HYK designed the study and drafted the manuscript. SMK, JHS, EHP, and DHL collected the clinical data. NK participated in the study design and coordination. All authors read and approved the final version of this manuscript.

## Pre-publication history

The pre-publication history for this paper can be accessed here:

http://www.biomedcentral.com/1471-230X/14/82/prepub

## References

[B1] Jung KW Park S Kong HJ Won YJ Boo YK Shin HR Park EC Lee JS Cancer statistics in Korea: incidence, mortality and survival in 2006–2007 J Korean Med Sci 2010 25 8 1113 1121 10.3346/jkms.2010.25.8.1113 20676319 PMC2908777

[B2] Lee BI Hong SP Kim SE Kim SH Kim HS Hong SN Yang DH Shin SJ Lee SH Park DI Kim YH Kim HJ Yang SK Kim HJ Jeon HJ Multi-Society Task Force for Development of Guidelines for Colorectal Polyp Screening, Surveillance and Management Korean guidelines for colorectal cancer screening and polyp detection Clin Endosc 2012 45 1 25 43 10.5946/ce.2012.45.1.25 22741131 PMC3363119

[B3] Leggett B Whitehall V Role of the serrated pathway in colorectal cancer pathogenesis Gastroenterology 2010 138 6 2088 2100 10.1053/j.gastro.2009.12.066 20420948

[B4] Snover DC Update on the serrated pathway to colorectal carcinoma Hum Pathol 2011 42 1 1 10 10.1016/j.humpath.2010.06.002 20869746

[B5] Nishihara R Wu K Lochhead P Morikawa T Liao X Qian ZR Inamura K Kim SA Kuchiba A Yamauchi M Imamura Y Willett WC Rosner BA Fuchs CS Giovannucci E Ogino S Chan AT Long-term colorectal-cancer incidence and mortality after lower endoscopy N Engl J Med 2013 369 12 1095 1105 10.1056/NEJMoa1301969 24047059 PMC3840160

[B6] Rex DK Ahnen DJ Baron JA Batts KP Burke CA Burt RW Goldblum JR Guillem JG Kahi CJ Kalady MF O'Brien MJ Odze RD Ogino S Parry S Snover DC Torlakovic EE Wise PE Young J Church J Serrated lesions of the colorectum: review and recommendations from an expert panel Am J Gastroenterol 2012 107 9 1315 1329 quiz 1314, 1330 10.1038/ajg.2012.161 22710576 PMC3629844

[B7] Rex DK Bond JH Winawer S Levin TR Burt RW Johnson DA Kirk LM Litlin S Lieberman DA Waye JD Church J Marshall JB Riddell RH U.S. Multi-Society Task Force on Colorectal Cancer Quality in the technical performance of colonoscopy and the continuous quality improvement process for colonoscopy: recommendations of the U.S. Multi-Society Task Force on Colorectal Cancer Am J Gastroenterol 2002 97 6 1296 1308 10.1111/j.1572-0241.2002.05812.x 12094842

[B8] Kaminski MF Regula J Kraszewska E Polkowski M Wojciechowska U Didkowska J Zwierko M Rupinski M Nowacki MP Butruk E Quality indicators for colonoscopy and the risk of interval cancer N Engl J Med 2010 362 19 1795 1803 10.1056/NEJMoa0907667 20463339

[B9] Hetzel JT Huang CS Coukos JA Omstead K Cerda SR Yang S O'Brien MJ Farraye FA Variation in the detection of serrated polyps in an average risk colorectal cancer screening cohort Am J Gastroenterol 2010 105 12 2656 2664 10.1038/ajg.2010.315 20717107

[B10] Liang J Kalady MF Appau K Church J Serrated polyp detection rate during screening colonoscopy Colorectal Dis 2012 14 11 1323 1327 10.1111/j.1463-1318.2012.03017.x 22390284

[B11] Kahi CJ Li X Eckert GJ Rex DK High colonoscopic prevalence of proximal colon serrated polyps in average-risk men and women Gastrointest Endosc 2012 75 3 515 520 10.1016/j.gie.2011.08.021 22018551

[B12] Zauber AG Winawer SJ O'Brien MJ Lansdorp-Vogelaar I van Ballegooijen M Hankey BF Shi W Bond JH Schapiro M Panish JF Stewart ET Waye JD Colonoscopic polypectomy and long-term prevention of colorectal-cancer deaths N Engl J Med 2012 366 8 687 696 10.1056/NEJMoa1100370 22356322 PMC3322371

[B13] Singh H Nugent Z Demers AA Kliewer EV Mahmud SM Bernstein CN The reduction in colorectal cancer mortality after colonoscopy varies by site of the cancer Gastroenterology 2010 139 4 1128 1137 10.1053/j.gastro.2010.06.052 20600026

[B14] Baxter NN Warren JL Barrett MJ Stukel TA Doria-Rose VP Association between colonoscopy and colorectal cancer mortality in a US cohort according to site of cancer and colonoscopist specialty J Clin Oncol 2012 30 21 2664 2669 10.1200/JCO.2011.40.4772 22689809 PMC3413278

[B15] Brenner H Chang-Claude J Seiler CM Rickert A Hoffmeister M Protection from colorectal cancer after colonoscopy: a population-based, case–control study Ann Intern Med 2011 154 1 22 30 10.7326/0003-4819-154-1-201101040-00004 21200035

[B16] Rex DK Can we fix colonoscopy?…Yes! Gastroenterology 2011 140 1 19 21 10.1053/j.gastro.2010.11.025 21110966

[B17] Rex DK Petrini JL Baron TH Chak A Cohen J Deal SE Hoffman B Jacobson BC Mergener K Petersen BT Safdi MA Faigel DO Pike IM ASGE/ACG Taskforce on Quality in Endoscopy Quality indicators for colonoscopy Am J Gastroenterol 2006 101 4 873 885 16635231 10.1111/j.1572-0241.2006.00673.x

[B18] Yamauchi M Morikawa T Kuchiba A Imamura Y Qian ZR Nishihara R Liao X Waldron L Hoshida Y Huttenhower C Chan AT Giovannucci E Fuchs C Ogino S Assessment of colorectal cancer molecular features along bowel subsites challenges the conception of distinct dichotomy of proximal versus distal colorectum Gut 2012 61 6 847 854 10.1136/gutjnl-2011-300865 22427238 PMC3345105

[B19] Yamauchi M Lochhead P Morikawa T Huttenhower C Chan AT Giovannucci E Fuchs C Ogino S Colorectal cancer: a tale of two sides or a continuum? Gut 2012 61 6 794 797 10.1136/gutjnl-2012-302014 22490520 PMC3345045

[B20] Brenner H Chang-Claude J Seiler CM Hoffmeister M Interval cancers after negative colonoscopy: population-based case–control study Gut 2012 61 11 1576 1582 10.1136/gutjnl-2011-301531 22200840

